# The effect of alpha linolenic acid on tracheal responsiveness, lung inflammation, and immune markers in sensitized rats

**DOI:** 10.22038/ijbms.2019.27381.6684

**Published:** 2019-03

**Authors:** Mahsa Kaveh, Naeima Eftekhar, Mohammad Hossein Boskabady

**Affiliations:** 1 Department of Biology, Science and Research Branch, Islamic Azad University, Tehran, Iran; 2 Department of Biology, Faculty of Science, Ferdowsi University of Mashhad, Iran; 3 Neurogenic Inflammation Research Center, Mashhad University of Medical Sciences, Mashhad, Iran; 4 Department of Physiology, School of Medicine, Mashhad University of Medical Sciences, Mashhad, Iran

**Keywords:** Asthma, Alpha linolenic acid, Inflammatory markers, Sensitized rats, Th1/Th2 balance, Tracheal responsiveness

## Abstract

**Objective(s):**

The effects of alpha linolenic acid (ALA) on tracheal responsiveness (TR), total protein (TP), phospholipase A2 (PLA2), immunoglobulin E (IgE), interleukin 4 (IL-4), interferon gamma (INF-γ) level and INF-γ/IL4 ratio in bronchoalveolar lavage fluid (BALF) of sensitized rats were examined.

**Materials and Methods:**

TR to methacholine and ovalbumin (OA), BALF levels of TP, PLA2 and IgE as well as IL-4, INF-γ and INF-γ/IL4 ratio were measured in control group (non-sensitized, group C), sensitized rats to OA (group S), S groups treated with two concentrations of ALA and dexamethasone group.

**Results:**

TR to methacholine and OA, BALF levels of TP, PLA2, IgE and IL-4 were significantly increased but BALF level of INF-γ and INF-γ/IL4 ratio decreased in group S compared to group C (*P*<0.001 for all cases). Treated S groups with dexamethasone and both concentrations of ALA lead to significant decrease in TR to methacholine and OA, BALF levels of TP, PLA2, IgE and IL-4 compared to group S (*P*<0.001 for all case). The effects of all concentrations of ALA on INF-γ, IL-4 and INF-γ/IL4 ratio and also the effect of its highest concentration on TP and IgE level were significantly higher than dexamethasone treatment (*P*<0.001 for all cases).

**Conclusion:**

Results showed an immune modulatory effect of the ALA that increased INF-γ, INF-γ/IL4 ratio (as an index of Th1/Th2) and decreased IL-4 in sensitized rats. ALA also showed preventive effect on inflammatory markers and tracheal responsiveness in sensitized animals comparable to the effect of dexamethasone.

## Introduction

Asthma is a chronic lung inflammatory (1) with airway remodeling that associate with airway hyper-responsiveness (AHR) to pharmacological agonists and other stimuli (1). AHR is defined as airways constriction to small stimuli which do not induce airway constriction in normal subjects (2) which closely related to airway inflammation as the main underlying mechanisms of the disease (3).

In asthma, activated inflammatory cells, release phospholipase A_2_ (PLA_2_), (4) which resulted to synthesis of eicosanoids that play an important role in inflammatory process (4). Increased serum and bronchoalveolar lavage fluid (BALF) level of PLA_2_ (5) and total protein was shown in subjects with occupational asthma (6). 

Airway inﬂammation in asthma is due to several inﬂammatory cells activation (7). In asthma, T helper 2 (Th2) is over-activated which its cytokines cause airway inﬂammation and mucus hyper-secretion (8). Th1 can inhibit Th2 responses and increased Th1/Th2 balance could be a treatment option of asthma (9). In allergic disease such as asthma and allergic rhinitis, interferon gamma/interleukin 4 (IFN-γ/IL-4) cytokines or Th1/Th2 balance shifted toward IL-4 or Th2 lymphocyte (10). Th2 interleukins such as IL-4 and interleukin 5 (IL-5), leads to immunoglobulin E (IgE) production and inducing of the growth of mucosal-type mast cells which can resulted in allergic response (11).

Alpha linolenic acid (ALA), an 18-carbon, essential omega-3 polyunsaturated fatty acid (PUFA) showed various pharmacological effects, such as anti-inflammatory (12-15), analgesic (16, 17), antibacterial (18), antimicrobial (19), antioxidant (20, 21) and neuro-protective properties (22-24). The relaxant effect of ALA on skeletal muscle (25, 26) and its effect on Th1/Th2 balances were demonstrated, suggesting its possible treatment effect in asthma which associated with decreased Th1/Th2 balance (27, 28). Therefore, in the present study, the effects of oral ALA on total protein, PLA_2_ and IgE level of lung lavage as well as Th1/Th2 balance and tracheal responsiveness in sensitized rats were examined.

## Materials and Methods

***Animals and studied groups***

Twenty six male Wistar rats (weighted 220±50 g) were purchased from Animal House, School of Medicine, Mashhad University of Medical Sciences and were kept in an animal cage in an animal room with clean filtered air (Maximiser, Thorens Caging System Inc, Hazleton, PA, U.S.A) at 22±2 ºC on a 12 hr light/dark cycle and water and food available *ad libitum* (29). Animals were randomly divided in five groups according to the previous study (30) and Table 1.

***Animal sensitization method***

**Figure 1:**
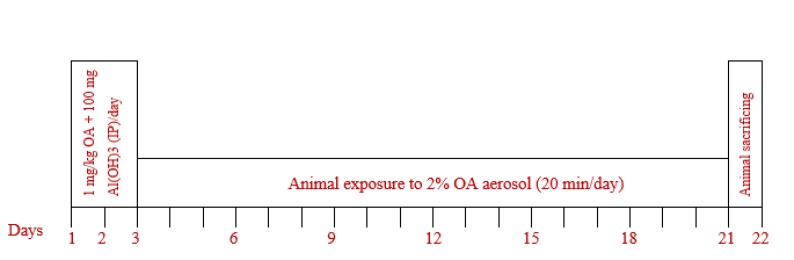
Method of rat sensitization. Ovalbumin (OA), 1 mg/kg+100 mg Al(OH)_3_ as adjuvant in 0.9% sterile saline were intraperitonaly (IP) injected on days 1, 2 and 3. Then rats were exposed to 1% OA aerosol for 20 min on days 6, 9, 12, 15, 18 and 21 with animal normal-breathing. OA aerosol was produced by a DeVilbiss PulmoSonic nebulizer (DeVilbiss Health Care Ltd, Feltham, U.K) with an air flow of 8 lit/min in a 0.8 m^3^ chamber

Sensitization of rats was done by intra-peritoneal injections of ovalbumin (OA) and Al(OH)_3_ and their exposure to OA aerosol according to previous studies (31, 32) as shown in Figure 1. Animal handeling were performed in compliance with the rulings of the Institute of Laboratory Animals Resources Commission on Life Sciences and the study was approved by the ethical Committee of the Mashhad University of Medical Sciences.

***BALF preparation***

BALF was prepared exactly as described in previous study (33).

***Measurement of BALF levels of total protein*** *,* ***PLA***_2_ ***, IgE, IL-4 and IFN-γ***

Total protein, PLA_2_ and IgE levels were measured using the enzyme-linked immunosorbent assay (ELISA) sandwich method according to the manufacturer protocol with photometric method as previously described (34).

***Tracheal tube preparations and measurement of racheal responsiveness (TR) to methacholine and ovalbumin***

Tracheal tube containing 5–6 cartilaginous was prepared, mounted in organ bath containing Krebs-Henseliet solution (KHS) and equilibrated for one hour exactly as previously described (35). 

TR to methacholine was measured by performing cumulative log concentration–response curves to methacholine hydrochloride (Sigma, purity; 98%) and calculation of the effective concentration of methacholine, causing 50% of maximum response (EC_50_) according the previously described method (35). 

TR to OA was measured as its contractile response 10 min after producing 0.2% OA concentration in the organ bath according to the previously described method (35). 

***Statistical analysis***

The results were quoted as means±SEM. Comparison of the data among different groups as well as those of two concentrations of ALA were performed using one way analysis of variance (ANOVA) with Tukey-Kramer’s post-test by InStat (GraphPad Software, Inc, La Jolla, USA). *P* values less than 0.05 was considered as statistical significance.

## Results

**Figure 2:**
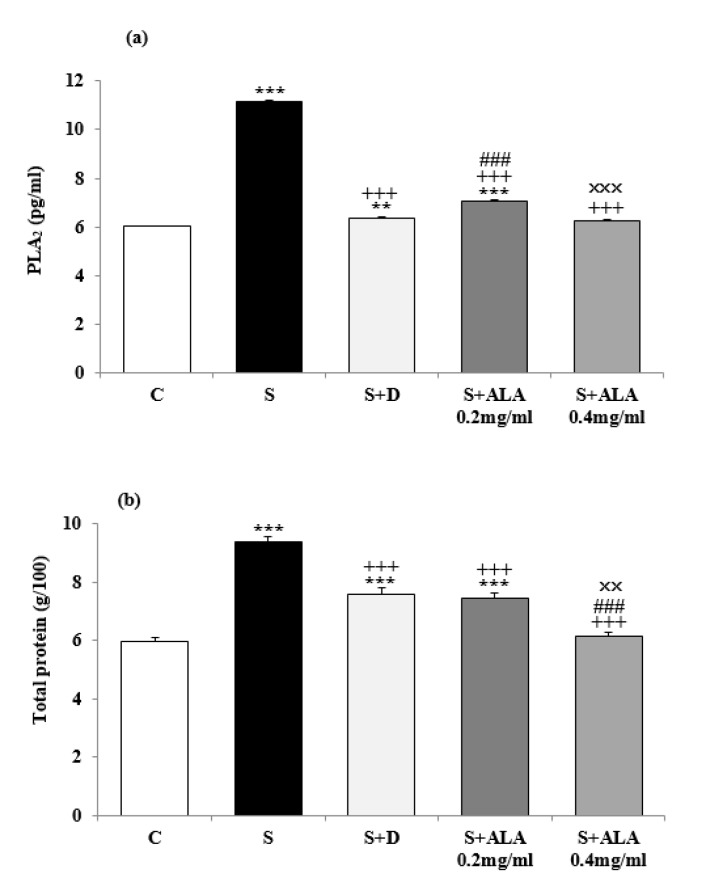
BALF levels (mean±SEM) of PLA2 (a) and total protein (b) in control rats (C), sensitized animals (S), S treated with dexamethasone (S+D) and two concentrations of alpha linolenic acid (S+ALA), (n=4 for ALA treated groups and n=6 for other groups)

Significantly higher PLA_2_ and total protein levels in BALF were observed in group S compared to group C (*P*<0.001 for both cases, Figure 2) but their levels were significantly decreased in treated animals with both concentrations of ALA and dexamethasone compared to group S (*P*<0.001 for all cases, Figure 2). However, BALF levels of PLA_2_ and total protein in treated groups with dexamethasone and low concentration of ALA were significantly higher than group C (*P*<0.01 to* P*<0.001, Figure 2). The effect of low concentration of ALA on PLA_2_ level was significantly lower (*P*<0.001, Figure 2) but the effect of its high concentration (0.4 mg/mL) on total protein (TP) was significantly higher than dexamethasone (*P*<0.001, Figure 2). The effect of high ALA concentration on BALF levels of PLA_2_ and TP were significantly higher than the effect of its low concentration (0.2 mg/mL), (*P*<0.001 and *P*<0.01 for PLA_2_ and TP respectively, Figure 2). 

**Figure 3:**
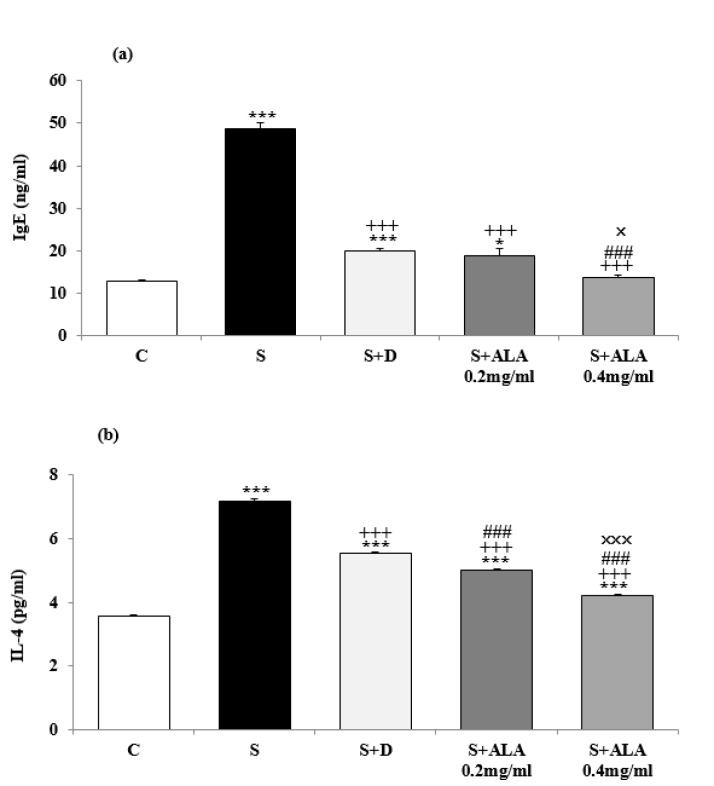
BALF levels (mean±SEM) of IgE (a) and IL-4 (b) in control rats (C), sensitized animals (S), S treated with dexamethasone (S+D) and two concentrations of alpha linolenic acid (S+ALA), (n=4 for ALA treated groups and n=6 for other groups)

**Figure 4:**
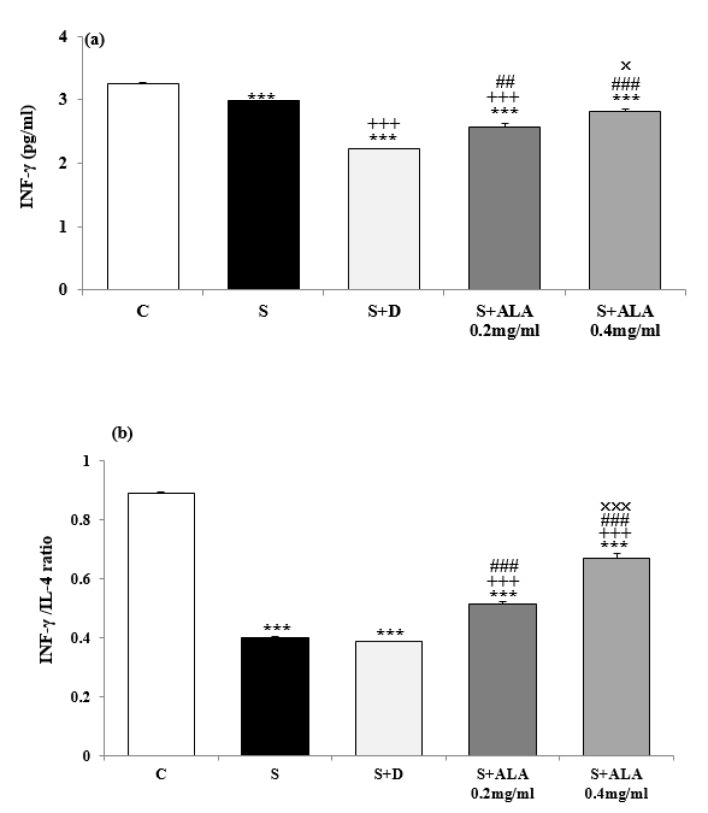
BALF levels of INF-γ (a) and INF-γ/IL-4 ratio (b) (mean±SEM) in control rats (C), sensitized animals (S), S treated with dexamethasone (S+D) and two concentrations of alpha linolenic acid (S+ALA), (n=4 for ALA and n=6 for other groups)

BALF level of IL-4 and IgE in group S were significantly higher but the level of IFN-γ and INF-γ/IL-4 ratio were significantly lower than group C (*P*<0.001 for all cases, Figures 3 and 4). IL-4 and IgE levels in treated groups with dexamethasone and both concentrations of ALA and IFN-γ levels in treated groups with dexamethasone and low concentration of ALA were significantly decreased compared to group S (*P*<0.001 for all cases, Figures 3 and 4). However, INF-γ/IL-4 ratio in treated groups with both concentrations of ALA were significantly increased compared to group S (*P*<0.001 for both cases, Figure 4). The effect of both concentrations of ALA on IL-4, IFN-γ and INF-γ/IL-4 ratio as well as its high concentration on IgE level were significantly higher than the effect of dexamethasone (*P*<0.001 for all cases, Figures 3 and 4). However, BALF levels of IgE and IL-4 were significantly higher but values of IFN-γ and INF-γ/IL-4 ratio were significantly lower in all treated groups than group C (*P*<0.001 for all cases, Figures 3 and 4). 

The effect of high ALA concentration (0.4 mg/ml) on BALF levels of IgE, IL-4, IFN-γ and INF-γ/IL-4 ratio were significantly higher than the effect of its low concentration (0.2 mg/ml), (*P*<0.05 to *P*<0.001, Figures 2-4). 

**Figure 5:**
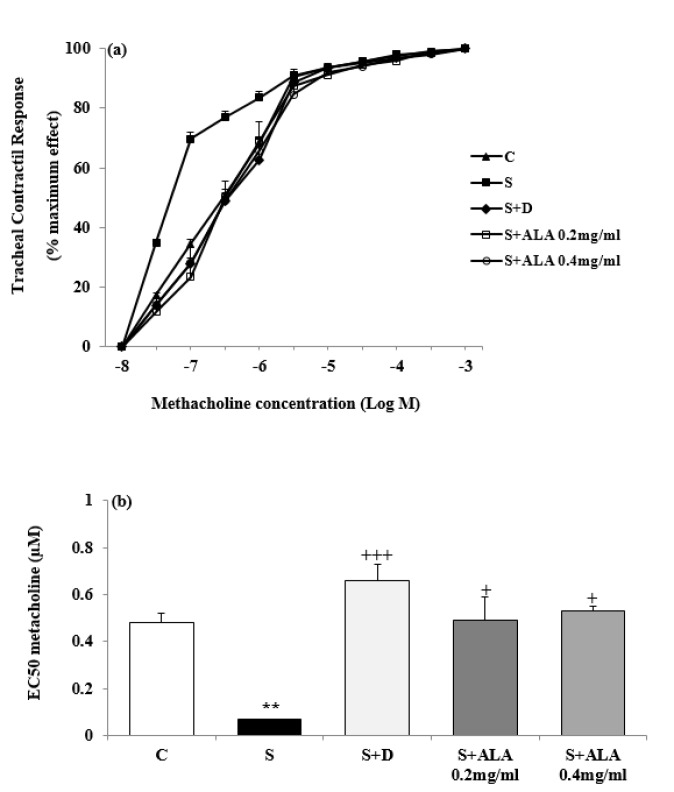
Cumulative log concentration-response curves of methacholine induced contraction of isolated trachea (a) and values (mean±SEM) of EC_50_, (the effective concentration of methacholine, causing 50% of maximum response) (b) in control rats (C), sensitized animals (S), S treated with dexamethasone (S+D) and two concentrations of alpha linolenic acid (S+ALA), (n=4 for ALA and n=6 for other groups)

**Figure 6:**
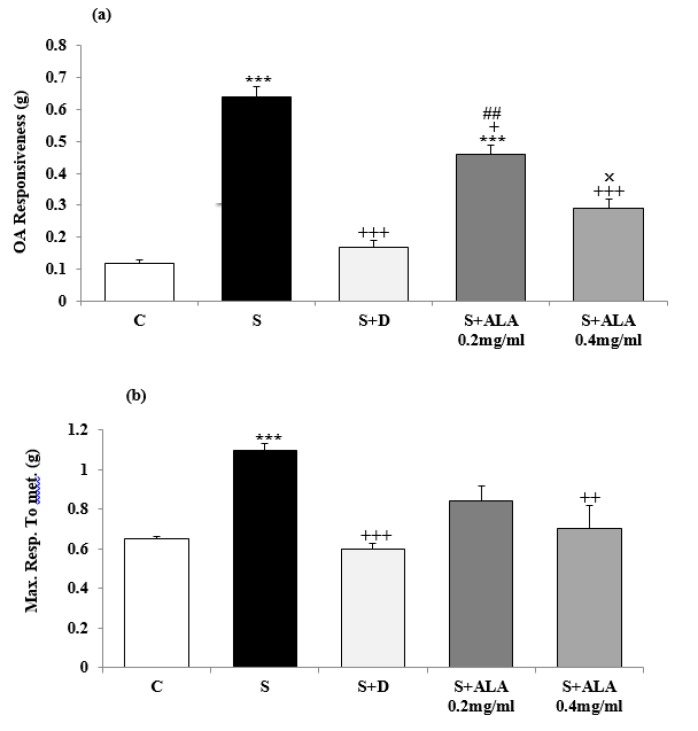
Values (mean±SEM) of maximum response to methacholine (a) and tracheal response to ovalbumin (b) in control rats (C), sensitized animals (S), S treated with dexamethasone (S+D) and two concentrations of alpha linolenic acid (S+ALA), (n=4 for ALA and n=6 for other groups).

Concentration-response curves to methacholine in group S shifted to left compared to group C but in the treated groups with dexamethasone and both concentrations of ALA, right-ward shift of the curves were observed compared to group S (Figure 5). EC_50_ methacholine was significantly lower but maximum response to methacholine and TR to OA were higher in group S compared to group C (*P*<0.01 for EC_50_ and* P*<0.001 for maximum response and tracheal response to OA, Figures 5 and 6). However, EC_50_ and TR to OA in treated groups with dexamethasone and both concentrations of ALA as well as maximum response to methacholine in treated groups with dexamethasone and high concentration of ALA were significantly improved compared to the group S (*P*<0.05 to *P*<0.001, Figures 5 and 6). The effect of low concentration of ALA on TR to OA was significantly lower than dexamethasone (*P*<0.01, Figure 6). TR to OA in treated group with low concentration of ALA was still significantly higher than group C (*P*<0.001, Figure 6). The effects of high concentration of ALA (0.4 mg/ml) on tracheal response to OA were significantly higher than its low concentration (0.2 mg/ml), (*P*<0.05, Figure 6). 

## Discussion

In present study, BALF level of TP, PLA_2_, IgE and IL-4 were increased but, INF-γ and INF-γ/IL-4 ratio were decreased in sensitized compared to control animal. In addition, left-ward shift in concentration-response curve to methacholine, increased, maximum response to methacholine and decreased EC_50_ methacholine were also seen in sensitized animal which indicated increased non-specific TR. Increased TR to OA was also observed indicating specific airway hyper responsiveness in sensitized animals. All these results indicated sensitization process or induction of animal model of asthma in rat. Similar changes in inflammatory and immune markers and TR were also shown in previous studies using similar method of animal sensitization (36-38) which support the findings of the present study. 

Treatment of sensitized animals with dexamethasone and both concentrations of the ALA caused significant reduction in BALF levels of PLA_2_, TP, IL-4 and IgE. Moreover, treatment by both concentrations of ALA significantly increased INF-γ/IL-4 ratio compare to sensitized group. Treatment with ALA and dexamethasone significantly increased EC_50_ and decreased maximum response to methacholine which indicated reduction of non-specific airway responsiveness in sensitized animals due to ALA and dexamethasone treatment. TR to OA as specific airway responsiveness also significantly decreased in sensitized animals treated with ALA and dexamethasone. The effect of ALA on decrease BALF levels of PLA_2_ and TP in sensitized rats indicates the preventing effect of ALA on lung inflammation of sensitized rats which is the most prominent lung change in asthma.

Statistical differences between groups S, S+D and S+ALA versus group C; ** *P*<0.01, *** *P*<0.001. Statistical differences between groups S+D and S+ALA versus group; S +++ *P*<0.001

Statistical differences between groups S+ALA versus group S+D; ### *P*<0.001

Statistical differences between groups S+ALA 0.2 mg/ml versus S+ALA 0.4 mg/ml; ×× *P*<0.01, ××× *P*<0.001. The statistical comparisons were made using one way analysis of variance (ANOVA) with Tukey-Kramer’s post-test

Statistical differences between groups S, S+D and S+ALA versus group C; * *P*<0.05, *** *P*<0.001. Statistical differences between groups S+D and S+ALA versus group S; +++ *P*<0.001

Statistical differences between groups S+ALA versus group S+D; ### *P*<0.001

Statistical differences between groups S+ALA 0.2 mg/ml versus S+ALA 0.4 mg/ml; × *P*<0.05, ××× *P*<0.001. The statistical comparisons were made using one way analysis of variance (ANOVA) with Tukey-Kramer’s post-test

Statistical differences between groups S, S+D and S+ALA versus group C; *** *P*<0.001

Statistical differences between groups S+D and S+ALA versus group S; +++ *P*<0.001

Statistical differences between groups S+ALA versus group S+D; ## *P*<0.01, ### *P*<0.001

Statistical differences between groups S+ALA 0.2 mg/ml versus S+ALA 0.4 mg/ml; × *P*<0.05, ××× *P*<0.001. The statistical comparisons were made using one way analysis of variance (ANOVA) with Tukey-Kramer’s post-test

Statistical differences between groups S, S+D and S+ALA versus group C; ** *P*<0.01

Statistical differences between groups S+D and S+ALA versus group S; + *P*<0.05, +++ *P*<0.001. The statistical comparisons were made using one way analysis of variance (ANOVA) with Tukey-Kramer’s post-test

Statistical differences between groups S, S+D and S+ALA versus group C; *** *P*<0.001

Statistical differences between groups S+D and S+ALA versus group S; + *P*<0.05, ++ *P*<0.01, +++ *P*<0.001.

Statistical differences between groups S+ALA versus group S+D; ## *P*<0.01

Statistical differences between groups S+ALA 0.2 mg/ml versus S+ALA 0.4 mg/ml; × *P*<0.05

The statistical comparisons were made using one way analysis of variance (ANOVA) with Tukey-Kramer’s post-test

**Table 1:** Experimental animal groups

Groups	Definition	Dose	Abbreviated name	n
Control	Non-sensitized rats		C	6
Sensitized	Non-treated, OA sensitized rats		S	6
	Treated with alpha linolenic acid (ALA)	0.2 mg/ml 0.4 mg/ml	S+ALA 0.2 mg/ml S+ALA 0.4 mg/ml	4 4
	Treated with Dexamethasone	1.25 μg/ml	D	6

Alpha linolenic acid (Sigma-Aldrich, Darmstadt, Germany), and dexamethasone (Sigma, purity; 97%) were added in drinking water of animals during sensitization period. Each rat in all groups averagely drank 40 ml/day drinking water which wasn’t significant difference among different groups. The animals of groups C and S were given drinking water alone.

The suppressive effect of ALA on IL-4 but its INF-γ enhancement in the BALF of sensitized animals suggests the inhibitory effect of ALA on Th2 and stimulatory effect on Th1 helper cells. INF-γ/IL-4 ratio was also increased in sensitized rats treated with ALA which indicated increased Th1/Th2 balance due to ALA treatment in animal model of asthma. The effect of ALA treatment on TR to methacholine and OA showed that this agent is able to reduce both specific and non-specific airway responsiveness in an animal model of asthma. Previous studies also indicated inhibitory effect of ALA on the production of interleukin-1 and tumor necrosis factor (34). Anti-inflammatory (12-15) and immunomodulatory effects of ALA on lactating dairy cows (39) have been demonstrated previously. The effect of ALA on Th1/Th2 balances was also demonstrated (27,28). The effect of hydro-ethanolic extract of *Portulaca oleracea* containing ALA on Th1/Th2 balance in isolated human lymphocytes was also reported (40). All the above studies support the findings of the present study which may suggest its therapeutic value in asthma by anti-inflammatory, immunoregulatory and its effect on TR mechanisms.

Therapeutic effects of omega-3 PUFA in asthma and exercise-induced bronchoconstriction (EIB) was reported. In addition, fish oil supplementation, rich in omega-3 PUFA, reduced airway narrowing, medication use, and inflammatory mediators in non-atopic elite athletes with EIB (41). Significant decrease in human blood lymphocyte proliferation and delayed-type hypersensitivity response were also seen 6 weeks after administration of linseed oil (providing about 15 g ALA/day) added to a low-fat diet (total fat provided 29% energy) (42). ALA also caused significant reduction in BALF level of IgE probably due to decreased lymphocytes proliferation which was depends on the level of linoleic acid and the total PUFA content of the diet (43). The blood lipid-lowering and immune-modulatory effects of ALA in rats was also shown (44). High dose of ALA (approximately 15 g/day) can suppress human Th1-drived cytokines production (42) which is in line with the findings of the current study. Supplementation of the diet with fish-oil derived omega-3 PUFA (1.2–14 g/day) results in decreased lymphocyte proliferation, production of IFN-γ (45), PGE2 production and synthesize of eicosanoids by immune cells. Omega-3 fatty acids are therefore, able to reduce disease-promoting inflammatory responses (45).

In the present study, dexamethasone was used as a corticosteroid positive control drug. Previously also, dexamethasone showed the inhibitory effect on airway inflammation in asthmatic mice (46). The effect of ALA on most measured variables was higher or at least equal to the effect of dexamethasone in sensitized rats. The effects of high concentration of ALA on TP and IgE levels and its both concentrations on IL-4 and IFN-γ levels as well as IFN-γ/IL-4 ratio were significantly higher than dexamethasone. In addition INF-γ/IL-4 ratio was only increased due to ALA treatment. Therefore, ALA showed equal or higher anti-inflammatory and effect on TR but more specific effect on Th1/Th2 balance compared to dexamethasone in rat model of asthma.

Concentration-dependent effects of the ALA on most measured variables were observed in the current study. The effects of high concentration of ALA on BALF levels of TP, PLA_2_, IgE, IL-4, INF-γ, INF-γ/IL-4 ratio and tracheal response to OA were significantly higher than its low concentration. The concentration dependency effect of ALA was also a further evidence for it effect on various measured variables on an animal model of asthma. 

Various effect of* P. oleracea* containing ALA on respiratory system including its relaxant effect on the tracheal smooth muscle (47,48), antitussive effect (49), bronchodilatory effect on asthmatic airways (50) as well as the possible mechanism(s) of the relaxant effect of the plant on tracheal smooth muscle such as its stimulatory effect on β-adrenoceptors (51) and anticholinergic property of this plant (52) were also demonstrated previously.

## Conclusion

Therefore ALA may have therapeutic effect on asthma by both bronchodilatory as relieving drug and effect on airway inflammation, immunomodulation and airway responsiveness as preventive drug. However, further studies including clinical trials needed to prove this suggestion.
